# Antioxidant Potential of Hemp and Flax Fibers Depending on Their Chemical Composition

**DOI:** 10.3390/molecules23081993

**Published:** 2018-08-10

**Authors:** Malgorzata Zimniewska, Wanda Rozańska, Agnieszka Gryszczynska, Barbara Romanowska, Anna Kicinska-Jakubowska

**Affiliations:** Institute of Natural Fibers & Medicinal Plants, Wojska Polskiego 71B, 60-630 Poznan, Poland; wanda.rozanska@iwnirz.pl (W.R.); agnieszka.gryszczynska@iwnirz.pl (A.G.); barbara.romanowska@iwnirz.pl (B.R.); anna.jakubowska@iwnirz.pl (A.K.-J.)

**Keywords:** flax, hemp, antioxidant activity, phenolic acids, chemical analysis, HPLC, ATR-FTIR

## Abstract

Flax and hemp fibers are known as textile raw materials with pro-health properties. This paper presents results of research aimed at investigating the antioxidant activity of the fibers in order to explain a mechanism for the favorable influence of textiles made of these fibers when in contact with human skin. The study presents a new approach in evaluation of textile fibers in terms of their inherent pro-health properties. Antioxidant properties of flax and hemp fibers were tested with the use of FRAP and DPPH methods. The content of phenolic acids: syringic, sinapinic, *p*-coumaric and ferulic acid was determined with the use of HPLC. The results proved that the antioxidant activity of the tested fibers depends on a type and variety of fibrous plants, a method of fiber extraction and subsequent stages of preliminary processing of the fibers. Both decorticated flax and hemp fibers showed higher antioxidant activity in comparison to the fibers obtained with other extraction methods, while decorticated flax of different varieties presented the highest value of the FRAP. Wet methods of fiber extraction and processing caused reduction of phenolic acids content and significantly lower values of the FRAP and DPPH.

## 1. Introduction

The care for health of the society and improvement of life quality have become priorities in the 21st century and also key challenges for the development of economy and many fields of science. This observable trend in developing and implementing of pro-health solutions is mainly focused on nutrition, ecological, healthy food, and specific diets as well as on cosmetics containing antioxidants for inhibition of aging processes. However, the closest environment of a human body is clothing, which affects the organism and skin condition, feeling of comfort and which shapes physiological markers of the user. The development of human-ecology goes along with the above-mentioned needs to protect organisms exposed to action of external factors. Clothing, meant as interaction with the human body, is most often studied in terms of comfort of using and its barrier properties, in the case of protective wear. Clothing made of natural fibers is always recommended by doctors as the safest for patients: with allergic reactions, affected with skin diseases, undergoing immunosuppressive treatments and with other ailments, where therapy is linked with necessity to eliminate possible negative effects of external factors, including that of the clothing. Taking under consideration this added value of flax fibers, a study [1] was conducted on clothing developed for patients suffering from dermatological diseases, which confirmed positive influence of clothing on healthy and sick skin. The positive influence of linen clothing on comfort is well known, especially when worn under conditions of low or moderate physical effort.

Apart from properties ensuring comfort, such as high hygroscopicity, air permeability, absence of accumulation of electrostatic charge, flax and hemp show inherent antibacterial and antioxidant properties. The antioxidant properties of flax fibers extracted from different cultivars with the use of dew retting and water retting methods were presented for the first time by Zimniewska [2]. The author proved that the biological activity of the tested flax fibers was strongly related to the lignin content and to the phenolic acid content in their chemical composition. In particular, it was shown that dew retted fibers contained a higher amount of lignin and phenolic acids, hence they were characterized by better antioxidant properties in comparison to water retted fibers.

The aim of the current study was to investigate antioxidant activity of hemp fibers and to study further the flax fibers obtained after application of different technological processes. This is a completely novel approach in terms of evaluating properties of flax and hemp fibers, which has not been reported so far elsewhere. Even though the level of the FRAP and DPPH of flax and hemp fibers is significantly lower in comparison to active food ingredients or dietary supplements, the newly discovered inherent bioactive properties of the fibers, by direct contact with skin surface, can support reduction of reactive oxidant species occurring at the skin surface and act as an anti-aging agent [3], protect against the unfavorable effect of the environment and ultraviolet rays [4,5]. New knowledge explaining antioxidant activity of bast fibers allows for designing and developing textiles with functional and protective characteristics. Newly determined properties of flax and hemp fibers allow for selection of efficient applications of the fibers in order to ensure specific functionality of textile products. The presence of phenolic substances in flax fibers has been studied by a few researchers [6,7], but there is no analysis of fibers antioxidant activity.

This paper contains results of the study on investigation of hemp and flax antioxidant activity depending on fibrous plants variety and applied processes. Statistical analysis and Pearson’s linear correlation coefficients were calculated to determine the level of the results relationship.

## 2. Results and Discussion

### 2.1. Chemical Analysis

One of the main components of flax and hemp fibers is cellulose, which constitutes of about 64–84% of the fiber mass [8]. Other components present along the cellulose include hemicellulose, lignin, pectins, waxes and fats. Proper removal of the accompanying components from the fiber will affect its further application in either textile or other industries. In the case of using the fiber in the textile industry, the fiber should be characterized with higher cellulose content and lower content of accompanying components i.e., hemicellulose, lignin, pectin, waxes and fats.

Table 1 shows the percentage share of individual compounds in the tested types of fibers i.e., for hemp of the Beniko, Wojko, Tygra and Białobrzeskie fibers after water retting, for the Białobrzeskie hemp fiber after dew retting, decortication, osmotic degumming and water retting, as well as for flax fibers of the Modran, Nike and B14 IUNG varieties after different stages of technological processes.

The tests indicated that for flax varieties both the content of cellulose and the accompanying components depended on the cultivar and the applied technological process. However, water retting and cottonization resulted in the increase of the cellulose content and decrease of hemicellulose content in all the tested varieties. For the other components i.e., pectin, lignin, waxes and fats the effect of the variety and the processes was more varied.

In the case of water retted hemp the only significant differences in the chemical composition of the fiber were found for the pectin levels while for the remaining components their levels were similar.

In the fiber of the Białobrzeskie variety the highest removal of the substances accompanying cellulose was achieved with the water retting method.

The results of the lignin content tests (Table 1) showed that the hemp varieties differed in this respect by less than 1%. For the flax varieties that value ranged between 4 and 8.6% depending on the technological process of obtaining the fibers. However, comparing the technological processes for extracting flax fibers did not indicate for clear effect on the lignin levels in the fiber.

Apart from testing the typical components of fibrous flax presented Table 1, the study included evaluating the content of phenolic compounds in the flax and hemp fibers, i.e., syringic, *p*-coumaric, sinapinic and ferulic acids, which might be responsible for the antioxidative activity of the fiber. The results for the four acids are presented in Table 2.

Based on an effectively scavenging chain reaction and deleterious radicals and suppressing radiation induced oxidative reactions, phenolic acids serve for preserving the physiological integrity of cells exposed to both air and to impinging UV radiation [4,5].

It is known that ferulic acid is a component of a primary cell wall and is bonded with lignin and hemicellulose in plants [7]. Different authors report that, flax fibers contain about 0.4–4 wt% phenolic compounds, depending on the methods used for the extraction [9,10,11].

Akin et al. found only small amounts of ferulic acid in flax fibers. Nuclear magnetic resonance (NMR) spectrometry confirmed that the aromatics were present as lignin [12].

Extraction of flax bast tissue, which comprised fibers and cuticle/epidermis, with a series of organic solvents (i.e., hexane, propanol, methanol, and water) and analysis by reverse phase high pressure liquid chromatography (HPLC) and ^13^C-NMR indicated for a variety of aromatic constituents, including flavonoids and hydroxy-methoxy cinnamic acids [13]. The water extract from those flax samples contained a complex mixture of compounds, including sugars and aromatics. The phenolic-containing extracts inhibited cellulase and pectinase activities, suggesting a possible influence on retting enzymes if such compounds were released. The author concluded that the most likely source of aromatic compounds in their study was the cuticle of the bast layer, rather than the fiber.

Opposite to the conclusion by Gamble et al., the results of the current study confirmed presence of ferulic, *p*-coumaric, syringic acids and small amounts of sinapinic acid in the chemical composition of flax and hemp fibers. The study showed diversity of phenolic acids content in the tested fibers, which resulted from different chemical composition of the fibers extracted from different fibrous plant varieties.

It is well established that the presence of syringic acid is correlated with high antioxidant and antibacterial activity. Syringic acid is a naturally occurring *O*-methylated phenolic acid that can be enzymatically degraded by some bacteria as a source of methane or methanol. It is also a component of phenolic extracts from various plants that have antioxidant and pro-oxidant activities [14].

In this study, the highest content of syringic acid was found in the decorticated fibers of both types: hemp and Modran and B14 IUNG flax varieties. That might be attributed to the fact that the decortication is the only dry mechanical process, which does not create proper conditions for bacterial growth. The conditions of water retting, e.g., temperature at 32–33 °C in stagnant water enhance growing of anaerobic bacteria and result in intensive smell coming from volatile fatty acids (e.g., butyric acid) accompanying the process [15]. Several species of bacteria were identified and investigated during tank retting, of which spore-forming *Clostridium* spp. was shown to contribute considerably to pectin-degrading activity and, therefore, to retting [16]. The syringic acid can be enzymatically degraded by some bacteria [14] and because of that syringic acid content in water retted fibers is the lowest in comparison with the fibers extracted with the use of other methods. The processes of osmotic and wet degumming combined with ultrasound applied for flax fibers were conducted under conditions of continuous flow of clean water, what significantly limited bacterial growth. Contrary to the water retting, no smell was observed during the processes. In the case of dew retting mainly fungi take part in the decomposition of the woody part of fibrous plant stems, whereas the action of bacteria is limited. The content of syringic acid in these types of hemp fibers is lower than in the decorticated fibers and higher than in the water retted ones.

The second tested acid, e.g., sinapinic acid, occurred only in hemp fibers, the highest amount was detected in the decorticated Białobrzeskie hemp, as well as in the water retted Wojko and Tygra hemp.

Sinapinic acid (3,5-dimethoxy-4-hydroxycinnamic acid) is an orally bioavailable phytochemical, extensively found in spices, citrus and berry fruits, vegetables, cereals, and oilseed crops and is known to exhibit antioxidant, anti-inflammatory, anticancer, antimutagenic, anti-glycemic, neuroprotective and antibacterial activities [17]. In addition, sinapinic acid can exhibit the so-called photodimerization properties when illuminated with ultraviolet light [18].

Coumaric and ferulic acids are the main hydroxycinnamic acids in flax [7] and are good candidates to be bound to glycans but also to cell wall proteins [i.e., phenyl-phenyl or phenyl-ether linkages] [19].

The evaluation of chemical composition of flax and hemp fibers confirmed that *p*-coumaric and ferulic acid content depends on the plant variety and applied processes. Investigation of the water retted hemp fibers proved differences in the content of *p*-coumaric and ferulic acids in relation to the plant variety. In terms of the applied extraction method of hemp fiber, the osmotically degummed Białobrzeskie contained the highest content of *p*-coumaric acid. Both decorticated hemp and flax fibers showed the highest content of ferulic acid and *p*-coumaric acid (with the exception of Białobrzeskie). In most cases, the lowest levels of *p*-coumaric and ferulic acids were found in the fibers extracted with the use of water retting and wet degumming combined with ultrasound. This is due to the fact that ferulic acid is easily soluble in water and can be easily removed from the fibers by water during the retting process. Coumaric acid is poorly soluble in water and its removal could be only partial [2]. For the same reason, almost all the types of the decorticated fibers contained the highest amount of *p*-coumaric and ferulic acids because decortication is entirely dry mechanical process.

Among the hemp fibers the Wojko variety showed the highest content of lignins (Table 1) and phenolic acids (syringic and *p*-coumaric acids—see Table 2) as compared to the other varieties of hemp (Beniko, Tygra, Białobrzeskie). The Białobrzeskie variety was characterized by the lowest content of lignins (Table 1) and phenolic acids (Table 2); sinapinic and ferulic acids were not identified.

In terms of the extraction method, the Białobrzeskie hemp fiber extracted with the mechanical process of decortication was characterized by the highest content of lignins (Table 1) and phenolic acids: syringic acid, sinapinic acid and ferulic acid (Table 2).

In the case of the B14 IUNG flax fiber the decrease in phenolic acids (syringic acid, *p*-coumaric acid and ferulic acid) content was observed after subsequent stages of the technological process. In the case of Modran and Nike varieties, the content of the phenolic acids (syringic acid, sinapinic acid, *p*-coumaric acid) was not identified.

Therefore, this study on the hemp fibers showed a relation between the lignin levels in the fiber and the values of the phenolic acids, for which the increase in the lignin caused the growth of the phenolic acids content. That phenomenon is linked with the structure of the compounds, where in both compounds a phenol molecule is present. Yet, that link was not proved for the flax fiber, for which the findings were ambiguous.

### 2.2. Antioxidant Activity

The table below (Table 3) presents the antioxidant activity for the selected flax and hemp fibers after different methods of the fiber extraction and processing.

The test results of the antioxidant activity of hemp varieties assessed with the use of the FRAP method proved that the water retted Tygra variety had higher ability for Fe ion reduction from Fe^+3^ to Fe^+2^ in comparison to other varieties. The highest antioxidant activity of the Tygra variety was also observed in the DPPH method. The bioactivity of the Tygra fiber resulted from the highest content of the ferulic acid and high content of other phenolic acids in the fiber.

In the case of the Białobrzeskie fiber extracted with the use of different methods, the highest capacity for Fe ions reduction from Fe^+3^ to Fe^+2^ and inhibition of the radicals by the DPPH method were observed for the decorticated hemp fibers. It is related to the highest content of all four phenolic acids in the fiber.

As a consequence of the subsequent stages of the technological process (decortication, water degumming, cottonization) of the different varieties of flax fiber, the decreasing of Fe ion reduction from Fe^+3^ to Fe^+2^ and inhibition of the radicals by the DPPH method were observed.

The evaluation of the FRAP and DPPH of the tested hemp and flax proved that antioxidant activity of the fibers is strongly related to the variety of plants and the type of applied technological processes. However, the water retting and water degumming combined with ultrasound treatment negatively influenced fibers activity but the decortication process produced fibers characterized by the highest phenolic acids content and the highest values of the FRAP and DPPH.

When analyzing the FTIR and DPPH of all the tested fibers it should be concluded that flax fibers were characterized by better bioactivity in comparison to hemp fibers. The comparison is possible only for the decorticated fibers as different processes were applied for flax and other for hemp fibers. Among all the decorticated flax fiber samples, the Modran variety showed the highest FRAP parameter. This result is compatible to the results obtained by Zimniewska [2], where the study on different flax varieties subjected to two retting methods proved that the Modran variety showed the best antioxidant properties. It was found that the FRAP parameter determined for Modran variety extracted from stem with use of dew retting reached the highest value 225 μmol/L. This value cannot be directly compare to results obtained in this investigation due to different method of fiber extraction applied in previous study.

The hemp fibers obtained with use of different extraction methods and flax fibers after different stages of processing were characterized for lignin and phenolic acid contents. The correlation between lignin content, the FRAP and DPPH parameters and the content of ferulic, *p*-coumaric, syringic and sinapinic acids depending on the hemp varieties and the methods of obtaining the fibers are presented in Table 4. The same table shows the correlation in the case of the selected flax varieties depending on subsequent stages of processing. Some of the results were below the HPLC detection threshold, what did not allow for conducting the statistical analysis. The statistical analysis showed strong correlation between the variables, the fiber composition and its bioactivity.

The correlation coefficients are presented as linear functional relationships with the confidence intervals at 95%.

The Modran variety after subsequent processes showed the strongest correlation between phenolic acids content (ferulic, *p*-coumaric and syringic) and lignin as well as FRAP and DPPH determined for the fibers, even though the lignin content decreased after each stage of technological chain. This correlation is clear because the decorticated fibers always contain higher amounts of lignin resulting from high content of impurities. Each further process caused reduction of impurities and of lignin and other non-cellulosic substances. This regularity was observed also for B14 IUNG variety, however the correlation was not as strong as in the case of Modran. The highest correlation between the antioxidant activity and the content of syringic acid and slightly lower but still strong correlation for the ferulic acid content was found for the B14IUNG variety. For the flax NIKE variety, all the Pearson coefficients could be determined only for the ferulic acid and their values were lower in comparison with the other flax varieties.

In the case of the Białobrzeskie hemp extracted with use of different methods, the strongest correlation was found between the content of ferulic/syringic/sinapinic acids and values of the FRAP and DPPH as well as of lignin. The highest values of the Pearson correlation coefficient calculated for different varieties of hemp was observed for relationship between the ferulic acid and DPPH, *p*-coumaric acid and lignin content and the FRAP as well as for the sinapinic acid and DPPH and FRAP.

**Figure 1:**
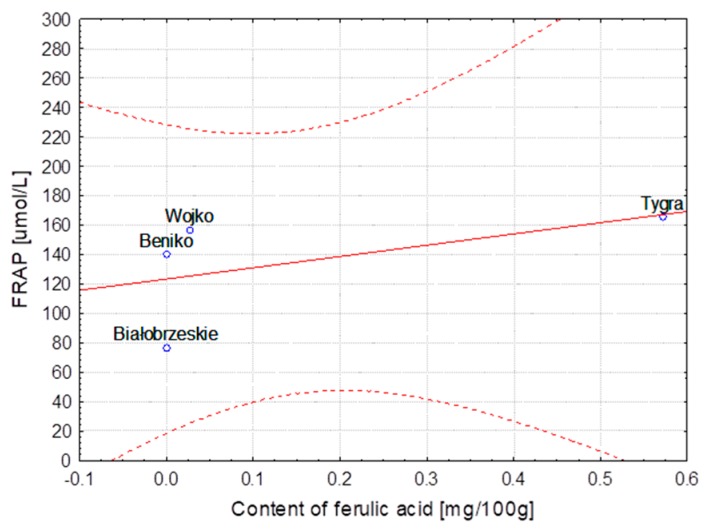
Method extraction of hemp—Dependence of FRAP [μmol/L] vs. content of ferulic acid [mg/100 g] (y = 87.057 + 64.243x; C = 95%).

**Figure 2:**
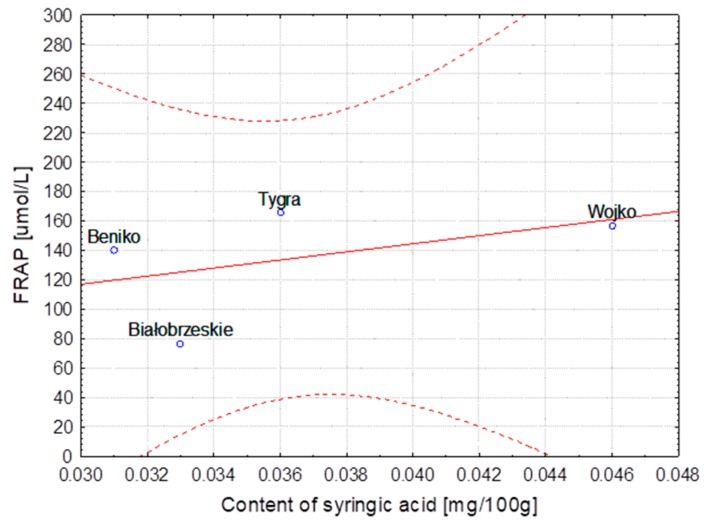
Method extraction of hemp—Dependence of FRAP [μmol/L] vs. content of syringic acid [mg/100 g] (y = 43.999 + 810.796x; C = 95%).

**Figure 3:**
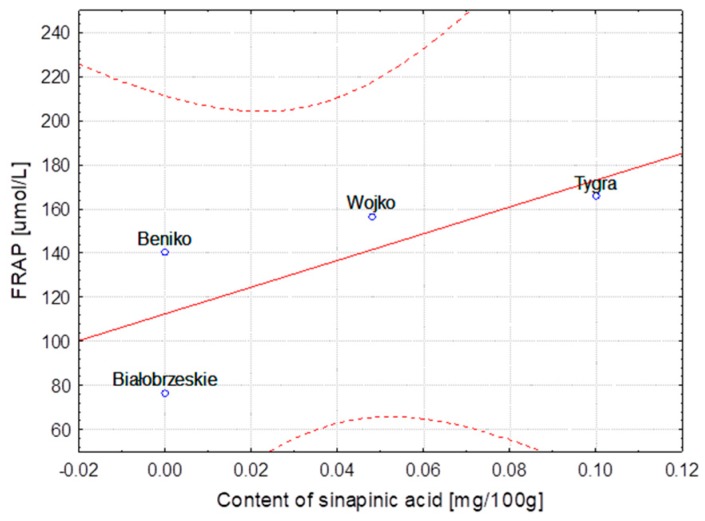
Method extraction of hemp—Dependence of FRAP [μmol/L] vs. content of sinapinic acid [mg/100 g] (y = 98.140 + 195.548x; C = 95%).

**Figure 4:**
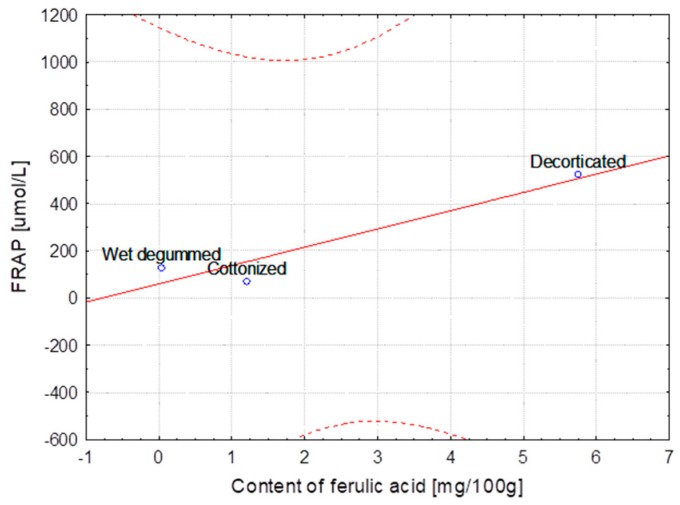
Variety of flax MODRAN—Dependence of FRAP [μmol/L] vs. content of ferulic acid [mg/100 g] (y = 60.187 + 77.556x; C = 95%).

**Figure 5:**
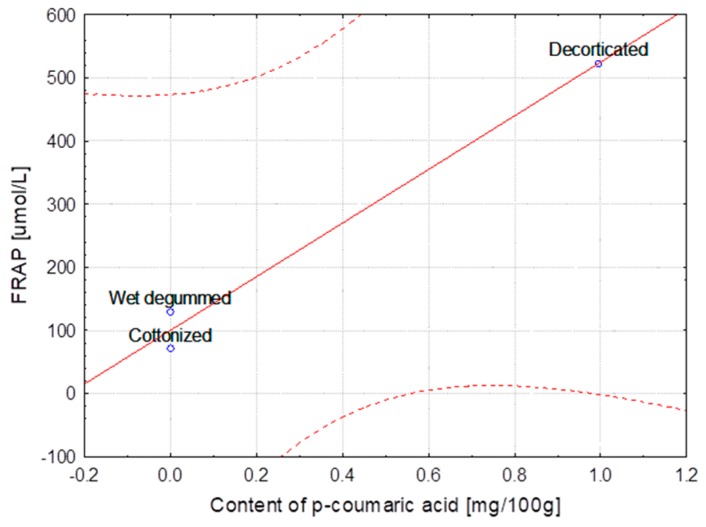
Variety of flax MODRAN—Dependence of FRAP [μmol/L] vs. content of *p*-coumaric acid [mg/100 g] (y = 100.070 + 425.055x; C = 95%).

**Figure 6:**
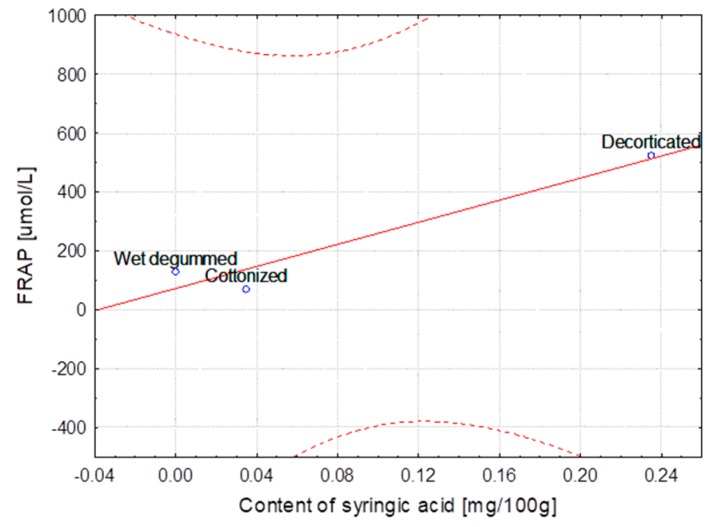
Variety of flax MODRAN—Dependence of FRAP [μmol/L] vs. content of syringic acid [mg/100 g] (y = 72.254 + 1875.476x; C = 95%).

The strongest correlation, determined for hemp fibers obtained with use of different extraction methods as well as for Modran flax fibers after different processing, are illustrated at Figure 1, Figure 2, Figure 3, Figure 4, Figure 5 and Figure 6.

### 2.3. ATR-FTIR Analyses of Fiber

**Figure 7:**
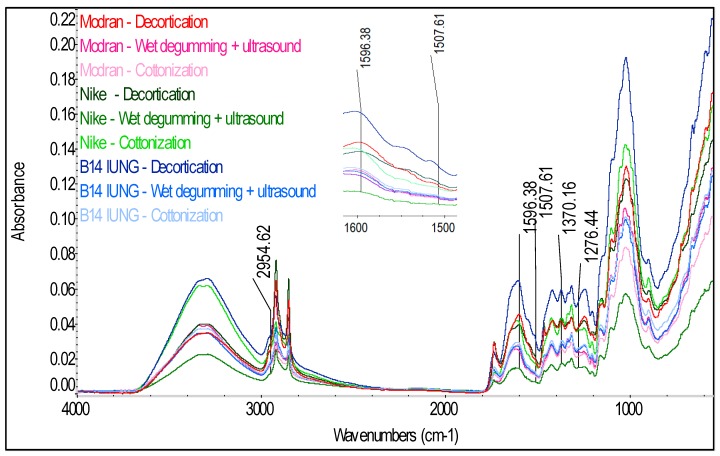
The Fourier transform infrared Attenuated Total Reflection spectra of the flax fibers of Modran, Nike and B14 IUNG variety after technological processes.

**Figure 8:**
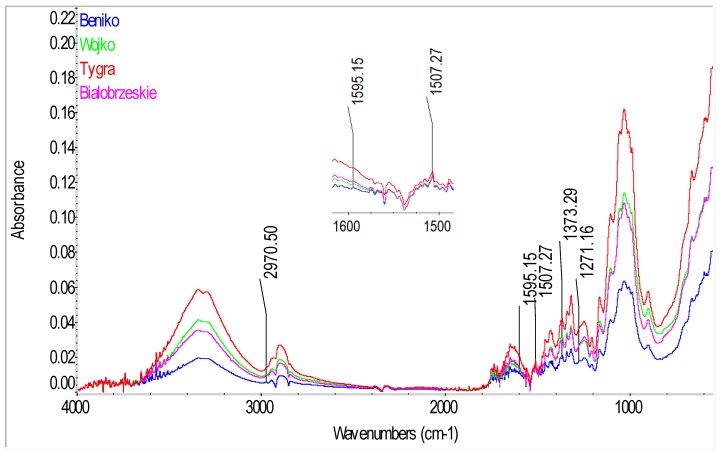
The Fourier transform infrared Attenuated Total Reflection spectra of the hemp fibers of Beniko, Wojko, Tygra and Białobrzeskie variety after warm water retting.

**Figure 9:**
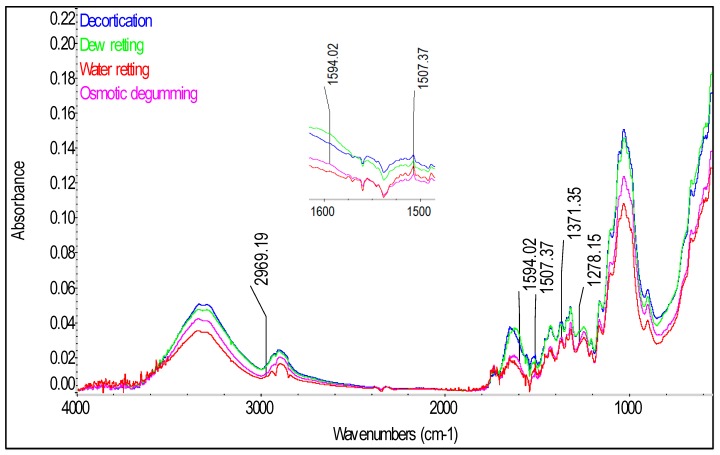
The Fourier transform infrared Attenuated Total Reflection spectra of the Białobrzeskie variety, hemp fiber after decortication, dew retting, water retting and osmotically degumming.

In order to identify the compounds present in the fiber and to confirm the presence of phenolic acids in the tested hemp and flax fibers, spectrophotometric analysis was conducted by a total internal reflection method with the use of an Attenuated Total Reflectance (ATR) attachment. The spectra of the tested fibers in infrared are presented in Figure 7 for the flax varieties, Figure 8 for hemp varieties and Figure 9 for Białobrzeskie hemp fiber, after different extraction methods, such as: decortication, dew retting, and water retting and osmotic degumming.

The infrared spectra of the tested fibers (Figure 7, Figure 8 and Figure 9) serve to identify the absorbance ranges that represent vibrations of such functional groups as: O-H, C=O, C=C, COO, C-H, CH_2_, CH_3_, COC [20,21]. The characteristics of the main absorbance spectra in the FTIR of the tested fiber are shown in the Table 5.

The analysis of the FT-IR ATR spectra for all the tested fibers (Figure 7, Figure 8 and Figure 9) showed presence of common spectra within the following absorbance range: 893–896 (1,4-glycosidic bond), 910–1125 and 1244–1246 (C-O stretching), 1020–1028 and 1051 and 1156–1161 (C-O-C bending), 1201–1204 and 1280 (C-H bending), 1312–1314 (CH_2_ scissoring), 1332–1338 (OH bending), 1369–1371 (C-CH_3_ symmetrical), 1418–1420 and 1424–1426 (COO stretching), 1461–1463 and 1472–1473 (C-CH_3,_ C-CH_2_ deforming), 1461–1463 (O-H bending) and 1615–1645 (O-H stretching) for adsorbed water, 1506–1509 and 1545–1554 and 1588–1594 (C=C stretching) for the aromatic group, 1730–1736 (C=O stretching), 3336 and 3290 (O-H, stretching) cm^−1^ [21]. Those spectra differ only in the intensity of the signal between the tested fibers—Figure 7, Figure 8 and Figure 9.

Significant differences can be observed for the spectra in the absorbance range of 2800–3000 cm^−1^ (C-H, C-H_2_, C-H_3_ stretching), where for the tested fibrous flax varieties two distinct spectra were found at the ranges of 2915–2923 and 2841–2848 cm^−1^ while for the hemp varieties at the ranges of 2895–2897 and 2939 cm^−1^.

The analysis of the fiber structure showed that all the identified spectra were linked with the chemical components of the fiber i.e., cellulose, hemicellulose, lignin, pectin, waxes and fats. However, separation of individual compounds from the spectrum is impossible as the same functional groups are present in the substances.

Cellulose, hemicellulose and pectin all belong to the polysaccharides similar in terms of molecules bonded with glycosidic bonds within the absorbance range at 896 cm^−1^ [20,22,23]. In the cellulose, the molecules are bonded with (1,4)-β-glycosidic bonds, while in hemicellulose and pectin with (1,4)-β and (1,3)-β-glycosidic.

Lignin is a polymer, monomers of which are organic compounds derived from phenolic alcohols. The most characteristic spectra for he lignin are symmetrical stretching vibrations for the C=C bond, from the aromatic ring at the absorbance of 1593–1595 and 1507–1508 cm^−1^ [21]. The spectra characteristic for lignin are also stretching vibrations from C-H_3_ bond in the range of 2954–2970 cm^−1^, symmetrical deforming vibrations from C-H_3_ bonds at 1370–1373 cm^−1^ and bending vibrations for the C-H bond at 1271–1278 cm^−1^ [21]. Importantly, the signal from the bending vibration for the C-H group was observed only for the spectra of the dew retted flax fibers.

Due to the fact that the tested phenolic compounds such as: syringic, *p*-coumaric, sinapinic and ferulic acids are found in lignin in the fibers, they should be detected in the IR spectrum in the bands characteristic for the lignin, described in Table 2. The spectra (Figure 7, Figure 8 and Figure 9) of the tested fibers represent in detail the bands specific for lignin at the absorption of 1500–1600 cm^−1^, from C=C bonds for the phenolic group. The analysis of the spectra indicated that the highest intensity of the signal for the flax (Figure 7) were observed for the decorticated fibers and the lowest for the fibers after the wet-degumming combined with ultrasound. In the case of hemp varieties (Figure 8) the highest intensity of the signal was found for the Tygra fibers and the lowest for the Beniko fibers. The analysis of the hemp fiber spectra (Figure 9) showed that the processes carried out in water i.e., the wet degumming combined with ultrasound and the osmotic degumming, caused decreased signals for the C=C bond in the aromatic ring. The decortication method turned out to be the mildest, where both the lignin (Table 1) and phenolic compounds content (Table 2) was the highest, for both flax and hemp fibers. This dependence is also visible in the signals from C-H_3_ bond at 2954–1970 cm^−1^ and 1370–1373 cm^−1^ and from C-H bond at 1271–1278 cm^−1^. The band characteristic for pectin includes stretching vibrations from the carboxylic group COO bond at 1418 and 1424–1426 cm^−1^. 

The analysis of spectra (Figure 7, Figure 8 and Figure 9) of the three groups of the tested fibers showed that the highest signal intensity was observed for: the B14 IUNG decorticated flax, the water retted Tygra hemp and the decorticated Białobrzeskie hemp. The lowest intensity of the signals (Figure 7, Figure 8 and Figure 9) was observed for: the Nike flax after wet degumming, the water retted Beniko hemp and the water retted Białobrzeskie hemp.

## 3. Materials and Methods

### 3.1. Materials

The initial material for the experiment was flax and hemp straw of Polish varieties, from which the fiber was extracted. All the varieties of the tested fibrous plants came from the same growing season. The weather conditions and applied agrotechnical measures for all tested varieties of flax were similar. Also the conditions of hemp growing were controlled and were similar.

Flax fiber, the initial material for the investigation was straw of Modran, Nike and B14 IUNG varieties. The traditional Modran variety was cultivated at the Institute of Natural Fibers & Medicinal Plants Processing Plant LENKON (Steszew, Poland) whereas the flax of the traditional Nike variety (with overexpression of -1,3-glucanase) and the B14 IUNG variety were cultivated at the Institute of Soil Science and Plant Cultivation (IUNG) in Pulawy at the experimental farm in Jelcz-Laskowice (Poland).

Hemp fiber, the initial material for the investigation was hemp straw of Białobrzeskie, Beniko, Wojko and Tygra varieties cultivated at INF&MP Experimental Farm in Petkowo (Poland).

### 3.2. Methods of Fiber Extraction

The process of extracting the flax fiber from straw included the stage of mechanical decortication, carried out in a technological line for decorticating of bast fibers for the textile industry [24]. Decortication, commonly used for flax or hemp, allows for dry mechanical extraction of fibers from plant stalks without the retting process. In the following processing stage, the decorticated fiber in the form of reeled sliver was subjected to wet degumming process with ultrasounds (Institute for Sustainable Technologies, Radom, Poland) with the use of a prototype technological line. The process was carried under the following conditions: in a closed device with liquid flow at the maximum speed, in water of 30 °C, for 24 h. Further on, the process was conducted in an open degumming device (Institute for Sustainable Technologies, Radom, Poland) in water of 30 °C and with 25 and 35 kHz frequency of ultrasounds. Finally, a mechanical process of cottonization on a carding machine (Ekotex, Kowalowice, Poland) was carried out [25].

Hemp straw of all the varieties was subjected to the water retting at 31 °C for 120 h in a laboratory scale tank at the Institute of Natural Fibers & Medicinal Plants. Hemp fiber from the Białobrzeskie variety was obtained by the use of different fiber extraction methods: water retting, dew retting, mechanical decortication, and osmotic degumming. The water retting was conducted in a lab-scale tank with temperature of water at 32 °C for 74 h, the dew retting was conducted after pulling out the straw directly on the field in ambient weather conditions for 43 days. The osmotic degumming method is based on natural physical laws: water diffusion, osmosis and osmotic pressure to obtain the higher quality of fibers. The process was supported by ultrasound treatment [26,27].

The experiment material included:

- Flax: Modran, Nike, B14 IUNG varieties (decorticated fiber, wet degummed with ultrasounds and cottonized fiber)
- Hemp: Białobrzeskie (water retted, dew retted, decorticated, osmotically degummed fiber)
- Hemp: Beniko, Wojko, Tygra, Białobrzeskie (water retted fiber).

### 3.3. Antioxidant Activity

#### 3.3.1. Sample Preparation

Approximately 1 g of fiber was placed in a round-bottom flask. Then 25 mL of 40% ethanol was added and the sample was heated under reflux condenser for an hour. After that, the solution was cooled down and filtrated through a filter into a 25 mL volumetric flask. The sample was filled up with 40% ethanol to the volume of 25 mL.

#### 3.3.2. Chemicals

TPTZ (2,4,6-tris(2-pyridyl)-1,3,5-triazine), DPPH (2,2-diphenyl-1-picrylhydrazyl) were provided by Sigma Aldrich (Darmstadt, Germany). Sodium acetate, acetic acid, chloric acid, ferric chloride and phosphoric acid were purchased in POCh (Gliwice, Poland). Acetonitrile was provided by J.T. Baker (Phillipsburg, NJ, USA).

#### 3.3.3. Determination of the Ferric Reducing Antioxidant Power (FRAP)

For determination of antioxidant activity, the method developed by Benzie and Strain (1996) was adapted in the study [28]. The fiber extract activity towards ferric ions was measured at different levels of the extract concentration, which was prepared as dilution of the sample solution with 40% ethanol. Then 0.5–2.5 mL of the sample solution was filled up with 40% ethanol to a volume of 2.5 mL. The test solution was prepared in a tube with 3.0 mL of the FRAP solution, 0.1 mL of the sample solution (at different concentrations) and 0.3 mL of water. The test tubes were incubated for 4 min in water bath (37 °C). Then, the samples were cooled down and the absorbance was measured at λ = 593 nm by comparison with the compensation liquid. The compensation liquid was prepared in 3.0 mL of the FRAP solution, 0.1 mL of 40% ethanol and 0.3 mL of water. That procedure was followed by preparing samples with the extract. The samples were prepared simultaneously. Fresh FRAP solution was prepared by mixing 30 mM TPTZ (2,4,6-tris(2-pyridyl)-1,3,5-triazine) in 40 mM chloric acid, 300 mM acetate buffer (pH = 3.6) and 20 mM ferric chloride solution in 1:10:1 (*v*:*v*:*v*) ratio.

#### 3.3.4. Determination of the 2, 2-Diphenyl-1-picrylhydrazyl (DPPH) Radical Scavenging Activity

Methods of DPPH inhibition developed by Huang et al., Katalinic et al. and Qian and Nihorimbere were adapted to the sample [29,30,31]. Activity of the fiber extract towards free radical was measured at different levels of the extract concentration, which was prepared as a dilution of the sample solution with 40% ethanol. The amounts of 0.5–2.5 mL of the sample solution were filled up with 40% ethanol to the volume of 2.5 mL. The test solution was prepared in a tube with 3.9 mL of DPPH ethanolic solution (6 × 10^−5^ M) and 0.1 mL of the sample solution (in different concentrations). The test solution was mixed and kept in the dark. The absorbance of the test solution was measured after 30 min at λ = 515 nm by comparison with 40% ethanol.

### 3.4. Determination of Phenolic Acids

#### 3.4.1. Sample Preparation

Approximately 0.6 g of the fiber was placed in an Erlenmeyer flask and 15 mL of 70% ethanol was added. The sample was extracted in ultrasonic (Institute of Natural Fibers & Medicinal Plants, Poznan, Poland) bath for 30 min, cooled down and filtrated. The fiber was extracted one more time with the same procedure. Supernatants were combined and evaporated to dryness in a rotary evaporator (Institute of Natural Fibers & Medicinal Plants) in vacuum. The residue was dissolved with 2 mL of 70% ethanol and the solution was transferred to a 2.0 mL volumetric flask.

#### 3.4.2. Chemicals

Analytical standards, such as: gallic acid, *p*-coumaric acid, ferulic acid were purchased in ChromaDex (Irvine, CA, USA) and syringic acid, sinapinic acid and dihydroxybenzoic acid in Sigma Aldrich.

#### 3.4.3. HPLC-DAD Analyses

The method of bioactive compound separation by Liu et al. (2006) and Gryszczynska et al. (2015) was adapted to this research [32,33]. High performance liquid chromatography (1100 system, Agilent, Institute of Natural Fibers & Medicinal Plants) was used to detect the phenolic acids. Separation and identification of the phenolic acids in the samples was obtained with chromatographic analysis using the Zorbax Poroshell 120 SB-C18 column, 2.7 mm × 3.0 mm × 100 mm (Agilent). A mixture of two solutions was used as a mobile phase i.e., the A phase: 0.1% phosphoric acid and the B phase: acetonitrile, and a gradient elution was used for the separation. A flow rate was 0.8 mL/min, starting with 90% A phase (13 min—78% A phase, 14 min—60% A phase, 30 min—60% A phase), the column temperature was 40 °C, and injection was 100 µL. The peaks were identified by the addition of standard solutions, using retention time and UV–vis spectra (Institute of Natural Fibers & Medicinal Plants) for qualitative analyses. The quantification of these compounds was achieved using calibration curves prepared with pure compounds. The detection of the substances was done at 205 nm, 303 nm and 330 nm.

### 3.5. Chemical Analyses

The following chemical testing methods were applied for the fiber evaluation:

▪ Cellulose content (%) in flax and hemp fiber was measured according to the Polish Standard no. PN-92/50092. The cellulose content was measured by dissolving lignins and other substances present in the fiber with a mixture of acetylacetone and dioxane, acidified with hydrochloric acid.
▪ Hemicelluloses content (%) in the flax end hemp fiber was determined according to the Polish Standard BN-77/7529-02. The hemicellulose content was measured by dissolving the hemicellulose present in the fiber with a 1% solution of sodium hydroxide, filtering off the residue after dissolution, drying it and weighing. Then the hemicelluloses were calculated from the mass loss of the sample.
▪ Lignin content (%) was determined according to the Polish Standard BN-86/7501-11. The lignin content was measured by dissolving cellulose, hemicellulose and pectins with a mixture of concentrated sulfuric and ortho phosphoric acids, followed by draining off the remaining insoluble lignin.
▪ Pectin content (%) tests were conducted by a gravimetric method according to a method developed at INF&MP. The percent share of pectins was determined by dissolving them in ammonium citrate and then precipitated from the solution with calcium chloride and by measuring the weight of the calcium pectinate precipitated from the solution.
▪ Waxes and fats content (%) was measured according to the Polish Standard no. BN-86/7501-10. The percentage content of wax and fat substances was determined by extracting them with an organic solvent (petroleum ether) in a Soxhlet extractor (Institute of Natural Fibers & Medicinal Plants) and weighing the residues after vaporization of the solvent.

### 3.6. ATR-FTIR Analysis

Fourier transform infrared spectroscopy (FTIR) with an ATR attachment was performed with a iS10 model instrument (TA Instruments, Institute of Natural Fibers & Medicinal Plants). The spectrum of the released gases contained 32 scans per second at a resolution of 4 cm^−1^ within the range from 600 to 4000 cm^−1^.

### 3.7. Statistical Analyses

The statistical analysis was performed using the STATISTICA software (8, Institute of Natural Fibers & Medicinal Plants). Data was expressed as mean ± standard deviation (SD). The significant differences between the fibers were assessed by the one-way analysis of variance (ANOVA) and Tukey’s Honest Significant Difference (HSD) test, and *p* < 0.05 was considered as significant difference. The correlation approach was chosen for analysis of the quality of the models due to ability of identification of linear relationship between two variables. Pearson’s linear correlation coefficients were determined in order to evaluate the strength of interdependencies between the variables. In order to provide a more accurate substantive interpretation, the obtained results of the correlation coefficients in the form of the linear functional relations with the indication of confidence intervals at the level of 95% were presented.

## 4. Conclusions

The results of the study proved that flax and hemp fibers exhibit inherent antioxidant properties, diversity of which depends on the plant variety, method of fiber extraction and subsequent stages of the technological chain applied for the fiber processing.

The tested flax fibers showed higher antioxidant activity in comparison with the hemp fibers. The FRAP and DPPH parameters reached the highest values for both types of fibers extracted with the use of decortication. The antioxidant activity is strongly correlated with lignin and phenolic acids content in the fibers.

The highest ability to reduce Fe ions from Fe^+3^ to Fe^+2^ showed decorticated flax fibers of Modran variety. The subsequently applied processes caused reduction of the FRAP and DPPH values. This knowledge should be used in designing functional textiles able to support protection of human skin against reactive oxygen species and UV radiation.

## Figures and Tables

**Table 1:** Chemical composition of: water retted hemp fibers of Beniko, Wojko, Tygra and Białobrzeskie variety; hemp fiber of Białobrzeskie variety after dew retting, decortication, osmotically degumming and water retting and flax fibers of Modran, Nike and B14 IUNG variety after technological processes. Results are expressed as mean ± standard deviation (SD). Lower-case letters indicate significant differences at *p* ≤ 0.05 according to the Tukay’s HSD test.

Degumming Method	Variety	Content of:									
		Waxes and Fats (*n* = 3)		Pectin (*n* = 5)		Lignin (*n* = 3)		Cellulose (*n* = 3)		Hemicellulose (*n* = 3)	
		%	±SD	%	±SD	%	±SD	%	±SD	%	±SD
**HEMP**											
Water retting	Beniko	0.23 ^a^	0.01	1.47	0.09	2.81 ^a^	0.29	71.31 ^a^	1.32	15.03 ^a^	0.02
	Wojko	0.24 ^a^	0.04	0.67 ^a^	0.02	3.02 ^a^	0.31	72.53 ^a^	0.11	16.67	0.24
	Tygra	0.25 ^a^	0.04	0.56	0.00	2.78 ^a^	0.28	70.79 ^a^	0.13	15.00 ^a^	0.28
	Białobrzeskie	0.34	0.02	0.67	0.02	2.38 ^a^	0.22	72.03 ^a^	0.22	14.37	0.29
Decortication		0.47 ^a,b^	0.02	2.00	0.09	5.55	0.17	66.02 ^a^	0.46	21.25	0.05
Dew retting		0.56 ^a^	0.14	3.68	0.19	4.31 ^a^	0.04	66.16 ^a^	0.48	21.72	0.12
Water retting	Białobrzeskie	0.34 ^b^	0.02	0.67	0.02	2.38	0.22	72.03	0.22	14.37	0.29
Osmotic degumming		0.44 ^a,b^	0.04	2.82	0.22	4.03 ^a^	0.09	67.81	0.52	16.29	0.03
**FLAX**											
Decortication		1.26	0.00	4.62 ^a^	0.16	4.00 ^a^	0.16	68.89	1.91	29.35	0.16
Wet degumming + ultrasound	Modran	0.69	0.07	4.41 ^a^	0.50	4.20 ^a^	0.16	75.54 ^a^	1.18	19.62	0.15
Cottonization		0.97	0.10	4.72 ^a^	0.39	4.26 ^a^	0.15	73.51 ^a^	0.98	16.44	0.23
Decortication		1.47	0.07	4.11	0.38	8.60	0.30	64.57	0.85	29.38	0.08
Wet degumming + ultrasound	NIKE	0.76	0.00	3.56	0.27	4.46 ^a^	0.48	77.44	1.58	16.43	0.25
Cottonization		0.95	0.05	2.39	0.22	4.87 ^a^	0.51	74.25	0.20	13.84	0.06
Decortication		1.47 ^a^	0.07	4.11	0.38	8.60	0.30	64.57	0.85	29.38	0.08
Wet degumming + ultrasound	B14 IUNG	1.33 ^a^	0.01	5.43	0.28	6.69 ^a^	0.48	75.04	0.46	23.92	0.02
Cottonization		1.72	0.09	3.57	0.23	6.10 ^a^	0.01	72.20	0.47	20.41	0.09

^a,b^—represent the groups for which the mean values do not differ statistically at the assumed significance level. The mean values labelled with the same letter (^a^ or ^b^) do not differ statistically at (α = 0.05).

**Table 2:** Acid content in the flax and hemp fiber. Results are expressed as mean ± standard deviation (SD), *n* = 4. Lower-case letters indicate significant differences at *p* ≤ 0.05 according to the Tukay’s HSD test.

Degumming Method	Variety	Content of Acids:							
		Syringic [mg/100 g]		Sinapinic [mg/100 g]		*p*-Coumaric [mg/100 g]		Ferulic [mg/100 g]	
		Result	±SD	Result	±SD	Result	±SD	Result	±SD
**HEMP**									
Water retting	Beniko	0.031 ^a^	0.001	-* ^a^	-	0.722 ^a,b^	0.019	-* ^a^	-
	Wojko	0.046	0.001	0.048	0.002	0.741 ^a^	0.006	0.027 ^a^	0.001
	Tygra	0.036	0.001	0.100	0.003	0.695 ^b^	0.034	0.572	0.031
	Białobrzeskie	0.033 ^a^	0.001	-* ^a^	-	0.024	0.006	-* ^a^	-
Decortication		0.224	0.011	0.672	0.023	0.746	0.008	2.082	0.036
Dew retting		0.079	0.003	-* ^a^	-	0.717	0.008	0.039 ^a^	0.004
Water retting	Białobrzeskie	0.033	0.001	-* ^a^	-	0.024	0.006	-* ^a^	-
Osmotic degumming		0.094	0.003	-* ^a^	-	1.111	0.011	0.625	0.009
**FLAX**									
Decortication		0.235	0.008	-* ^a^	-	0.995	0.024	5.749	0.159
Wet degumming + ultrasound	Modran	-*	-	-* ^a^	-	-* ^a^	-	0.041	0.002
Cottonization		0.035	0.002	-* ^a^	-	-* ^a^	-	1.206	0.053
Decortication		-* ^a^	-	-* ^a^	-	-* ^a^	-	1.485	0.034
Wet degumming + ultrasound	NIKE	-* ^a^	-	-*	-	-* ^a^	-	0.054	0.001
Cottonization		-* ^a^	-	-* ^a^	-	-* ^a^	-	1.035	0.020
Decortication		0.125	0.060	-* ^a^	-	0.904	0.009	3.146	0.106
Wet degumming + ultrasound	B14 IUNG	0.052 ^a^	0.002	-* ^a^	-	0.027	0.008	2.525	0.106
Cottonization		0.040 ^a^	0.001	-* ^a^	-	0.756	0.034	1.736	0.045

-* not identified; ^a,b^—represent the groups for which the mean values do not differ statistically at the assumed significance level. The mean values labelled with the same letter (^a^ or ^b^) do not differ statistically at (α = 0.05).

**Table 3:** The antioxidative activity of different varieties of bast fibers depending on the extraction method. Results are expressed as mean ± standard deviation (SD), *n* = 3. Lower-case letters indicate significant differences at *p* ≤ 0.05 according to the Tukay’s HSD test.

Degumming Method	Variety	FRAP [μmol/L]		Inhibition of DPPH [%]	
		Result	±SD	Result	±SD
**HEMP**					
Water retting	Beniko	140.34	4.75	11.30 ^a^	0.92
	Wojko	156.75	2.31	10.04 ^a^	0.49
	Tygra	165.76	1.62	32.55	0.32
	Białobrzeskie	76.62	1.33	3.09	0.18
Decortication		230.22	1.55	18.03	0.63
Dew retting		124.09	1.93	5.31	0.25
Water retting	Białobrzeskie	76.62	1.33	3.09 ^a^	0.18
Osmotic degumming		93.71	0.69	3.94 ^a^	0.19
**FLAX**					
Decortication		523.00	2.08	33.85	0.17
Wet degumming + ultrasound	Modran	129.45	1.28	5.80	0.14
Cottonization		70.69	2.31	3.29	0.24
Decortication		519.75	2.69	29.76	0.15
Wet degumming + ultrasound	NIKE	140.79	1.16	7.64	0.16
Cottonization		78.84	1.59	5.10	0.36
Decortication		485.84	2.11	37.71	0.14
Wet degumming + ultrasound	B14 Iung	195.79	2.23	11.05	0.11
Cottonization		119.42	4.55	6.82	0.23

^a^—represent the group for which the mean values do not differ statistically at the assumed significance level. The mean values labelled with the same letter (^a^) do not differ statistically at (α = 0.05).

**Table 4:** The values of the Pearson correlation determined for all the varieties of the tested fibers, relationship between the content of ferulic/*p*-coumaric/syringic/sinapinic acids and lignin content/FRAP/inhibition of DPPH.

Tested Parameters	Values of the Pearson Correlation Coefficient			
	Content of Ferulic Acid	Content of *p*-Coumaric Acid	Content of Syringic Acid	Content of Sinapinic Acid
Hemp variety				
Lignin content	0.11	0.93	0.66	0.43
FRAP	0.54	0.96	0.46	0.72
DPPH	0.96	0.54	0.02	0.89
Method extraction of hemp				
Lignin content	0.81	0.67	0.91	0.76
FRAP	0.91	0.28	0.96	0.96
DPPH	0.94	0.21	0.97	0.99
Variety of flax—MODRAN				
Lignin content	−0.91	−0.98	−0.94	Not identified
FRAP	0.95	0.99	0.97	Not identified
DPPH	0.96	1.00	0.98	Not identified
Variety of flax—NIKE				
Lignin content	0.80	Not identified	Not identified	Not identified
FRAP	0.65	Not identified	Not identified	Not identified
DPPH	0.68	Not identified	Not identified	Not identified
Variety of flax—B14 IUNG				
Lignin content	−0.53	−0.89	−0.85	Not identified
FRAP	0.92	0.46	1.00	Not identified
DPPH	0.89	0.53	1.00	Not identified

**Table 5:** The characteristics of the main absorbance spectra in FTIR of flax fiber.

Bond	Vibration Type	Wavenumber [cm^−1^]	Remarks
O-H	Stretching	3100–3600	Cellulose, hemicellulose, lignin, pectin
C-H_3_	Stretching	2954–2970	Lignin
C-H, C-H_2_	Stretching	2915–2923; 2895–2897; 2841–2848	Cellulose, hemicellulose, lignin, pectins, waxes and fats
C=O	Stretching	1730–1736	Carboxylic acids, aldehydes, esters (pectin, lignin, waxes and fats)
O-H	Stretching	1615–1645	Adsorbed water
C=C Aromatic	Symmetrical Stretching	1593–1595; 1507–1508	Peaks characteristic of lignin
O-H and C-H_3_ and C-H_2_	Bending and Deforming	1461–1463 and 1461–1463; 1472–1473	Adsorbed water and Lignin and cellulose, hemicellulose, pectins, waxes and fats
COO	Stretching	1418–1420; 1424–1426	Acids (pectins)
C-H_3_	Symmetrical Deformation	1370–1373	Lignin
O-H	Bending	1332–1338	Cellulose, hemicellulose, lignin, pectin
CH_2_	Scissoring (bending)	1312–1314	Cellulose, hemicellulose
C-H	Bending	1271–1278	Peak characteristic for lignin
C-O	Stretching	1244–1246	Hemicellulose, pectins
C-H	Bending	1201–1204	Flax, hemp
C-O-C	Bending	1156–1161; 1051; 1020–1028	Cellulose, hemicellulose, pectin
C-O	Stretching	910–1125	Cellulose, hemicellulose, pectin
Β-Glycosidic bond	Stretching	893–897	Cellulose, hemicellulose, pectin
